# Impact of Comorbidities on Treatments and Outcomes of Systemic Sclerosis–Associated Pulmonary Arterial Hypertension

**DOI:** 10.1155/carj/5021789

**Published:** 2025-11-03

**Authors:** Lisa Lim, Dylan Hansen, Jessica Fairley, Maryam Tabesh, Laura Ross, Nava Ferdowsi, Gene-Siew Ngian, Diane Apostolopoulos, Joanne Sahhar, Lauren V. Host, Jennifer Walker, Gabor Major, Susanna Proudman, Wendy Stevens, Mandana Nikpour

**Affiliations:** ^1^Department of Rheumatology, St. Vincent's Hospital, Melbourne, Victoria, Australia; ^2^Department of Medicine, The University of Melbourne, Melbourne, Australia; ^3^Department of Rheumatology, Monash Medical Centre, Melbourne, Victoria, Australia; ^4^Department of Medicine, Monash University, Melbourne, Australia; ^5^Fiona Stanley Hospital, Perth, Western Australia, Australia; ^6^Rheumatology Unit, Royal Adelaide Hospital, Adelaide, South Australia, Australia; ^7^Flinders Medical Centre, Adelaide, South Australia, Australia; ^8^Flinders University, Adelaide, South Australia, Australia; ^9^Department of Rheumatology, John Hunter Hospital, New Lambton Heights, Newcastle, New South Wales, Australia; ^10^Faculty of Medicine, University of Newcastle, Callaghan, Newcastle, New South Wales, Australia; ^11^Discipline of Medicine, The University of Adelaide, Adelaide, South Australia, Australia; ^12^Department of Rheumatology, Royal Prince Alfred Hospital, Camperdown, Sydney, New South Wales, Australia; ^13^The University of Sydney Musculoskeletal Research Flagship Centre, Sydney, New South Wales, Australia; ^14^School of Public Health, Faculty of Medicine and Health, The University of Sydney, Sydney, New South Wales, Australia

## Abstract

**Aim:**

Treatment recommendations for systemic sclerosis–associated pulmonary arterial hypertension (SSc-PAH) have evolved from initial monotherapy to upfront combination therapy with agents, including endothelin receptor antagonists, phosphodiesterase-5 inhibitors and prostanoids. In the presence of comorbidities, such as heart and lung disease, some clinicians have favoured monotherapy due to concerns about worsening ventilation–perfusion mismatch. We sought to evaluate whether comorbidity burden impacts prescribing practices, quality of life and survival in SSc-PAH.

**Methods:**

We analysed prospectively collected data from participants recruited to the Australian Scleroderma Cohort Study (ASCS) between 2007 and 2024. Participants were included if they had PAH confirmed by right heart catheterisation. Data were collected on the presence of 12 comorbidities as defined by the Charlson Comorbidity Index (CCI), and prescription of PAH therapies, at PAH diagnosis and each subsequent annual visit. High morbidity was defined as a CCI score ≥ 4. With regard to prescribing practices, subgroup analysis was performed on two groups. The cardiac comorbidity group included patients with a diagnosis of angina, acute myocardial infarction, congestive cardiac failure or hypertension. The pulmonary comorbidity group included those with a diagnosis of chronic obstructive pulmonary disease or asthma. An additional subgroup of patients with SSc-related interstitial lung disease (ILD) was compared to those without ILD. Survival was evaluated using the Kaplan–Meier method and a multivariable Cox regression model.

**Results:**

Among 2004 patients within the ASCS, 238 patients with SSc-PAH were included (11.8%). SSc-PAH patients had significantly higher CCI scores (3.0 vs. 2.0, *p* < 0.001) and were more likely to have a high morbidity index (30.3% vs. 18.6% *p* < 0.001). Within the cohort of SSc-PAH patients, there were no significant differences between high and low morbidity patients with regard to clinical characteristics, autoantibody profile or internal organ manifestation. There was no difference in use of PAH medications between SSc-PAH patients with a low and high morbidity, with similar proportions receiving combination, monotherapy and no therapy, *p*=0.10. This was also the case in a subgroup analysis of those with cardiac comorbidity, pulmonary comorbidity or SSc-ILD. When comparing SSc-PAH patients with high morbidity to those without using K-M survival analysis, there was higher all-cause mortality (*p*=0.05). Univariable survival analysis showed no significant survival difference between SSc-PAH patients with high and low comorbidity burden. Combination therapy for PAH was associated with better survival compared to monotherapy (HR 0.60, 95% CI: 0.43–0.84, *p*=0.003).

**Conclusion:**

In this large cohort of SSc-PAH patients, the choice of treatments did not appear to differ based on high comorbidity burden or coexisting pulmonary or cardiac comorbidity, with a majority of SSc-PAH patients receiving combination therapy. Receiving combination therapy, irrespective of comorbidity status, improved survival in our cohort.

## 1. Introduction

Systemic sclerosis (SSc) remains the largest connective tissue disease contributor to Group 1 pulmonary arterial hypertension (PAH) and is distinctly different in prognosis and management when compared to idiopathic PAH. Historically, PAH was considered a disease primarily affecting young women. However, data from established registries worldwide indicate a shift in epidemiology, with the disease now increasingly recognised in older patients who have more comorbidities [1]. This evolving demographic highlights the need for tailored treatment approaches that account for the presence of coexisting conditions and high comorbidity burden [2].

PAH is a severe vascular complication of SSc characterised by progressive remodelling of the small pulmonary arterioles, ultimately leading to increased pulmonary vascular resistance (PVR) and elevated pulmonary artery pressure [3]. Without adequate treatment, PAH can result in right ventricular failure and remains a leading cause of mortality in patients with SSc [1, 4].

The treatment paradigm for SSc-associated PAH (SSc-PAH) has undergone significant changes over time. Current guidelines emphasise the importance of comprehensive risk assessment, often using validated tools to guide therapeutic decision-making [5]. In previous years, treatment strategies predominantly favoured initial monotherapy with endothelin receptor antagonists (ERAs) or phosphodiesterase-5 (PDE5) inhibitors, with subsequent escalation to combination therapy with prostanoids [6]. This is based on evidence showing better outcomes with combination therapy, including the AMBITION trial, wherein those treated with upfront tadalafil combined with ambrisentan had a lower risk of clinical failure events than patients treated with tadalafil or ambrisentan alone [7]. A recent analysis of the Comparative, Prospective Registry of Newly Initiated Therapies for Pulmonary Hypertension (COMPERA) registry reported a marked increase in the use of combination therapy for connective tissue disease-associated PAH, rising from 6% in 2010 to 44% in 2019 [8]. Historically, in the presence of comorbidities such as heart and lung disease, some clinicians have favoured monotherapy, typically with PDE5 inhibitors, due to concerns about worsening ventilation–perfusion mismatch [8].

In addition to PAH, many patients with SSc have significant comorbidities, which can complicate disease management and influence treatment decisions [9]. The Charlson Comorbidity Index (CCI), originally developed to predict survival based on chronic disease burden, is widely used to quantify comorbidities in population-based studies [10, 11]. Using data from the Australian Scleroderma Cohort Study (ASCS), we aimed to evaluate whether comorbidity burden, as assessed by the CCI, impacts the treatments that patients with SSc-PAH receive and their quality of life and survival.

## 2. Methods

Participants recruited to the ASCS between 2007 and June 2024 were included if they met the following criteria: (1) Fulfilment of the 2013 American College of Rheumatology/European League Against Rheumatism SSc classification criteria for SSc (2) and the presence of PAH confirmed by right heart catheterisation (RHC) diagnosed after the enrolment visit. Patients who were diagnosed with PAH prior to their first visit were excluded. PAH was defined according to the World Symposium of Pulmonary Hypertension (WSPH) definition as mean pulmonary artery pressure (mPAP) > 20 mmHg, pulmonary artery wedge pressure (PAWP) ≤ 15 mmHg and PVR > 2 Wood units [12]. For participants without PVR data, PAH was defined by a mPAP ≥ 25 mmHg and PAWP ≤ 15 mmHg; lack of PVR recording in some instances is due to the inclusion of PVR in PAH diagnostic criteria from 2013 onwards only. Data were collected annually. The ASCS received ethics approval from all participating sites, with St. Vincent's Hospital Melbourne serving as the coordinating centre (HREC-A 020/07). Written informed consent was obtained from all participants.

Patient characteristics were recorded at enrolment into the cohort and at the visit where PAH was diagnosed. The ASCS database includes annual records of demographics, clinical data and medication use. Patients in the ASCS are routinely screened annually for PAH, using a combination of transthoracic echocardiography (TTE), blood N-terminal pro-brain natriuretic peptide (NT-proBNP) level and pulmonary function testing for forced vital capacity (FVC) and diffusing capacity for carbon monoxide (DLCO). Interstitial lung disease (ILD) is diagnosed based on the presence of radiological features on high-resolution computed tomography (HRCT) of the chest. Congestive heart failure (CHF) was defined as left ventricular ejection fraction (LVEF) < 50% on TTE. Chronic kidney disease (CKD) is defined as a history of creatinine higher than 265 *μ*ml/L, dialysis or renal transplantation. Ischaemic heart disease (IHD) is defined as patient-reported ischaemic chest pain or abnormal coronary angiography. Clinical examination features were recorded as present or absent by the study physician at each visit, including digital ulcers and calcinosis. Disease manifestations were considered present if they occurred at any point after SSc diagnosis.

Combination therapy was defined as the initiation of two or more classes of pulmonary hypertension therapy at any point after PAH diagnosis. Classes of PAH therapy include PDE5 inhibitors, ERAs and prostanoids.

A modified nineteen-item version of the CCI as per previous publications from our group was used, with data for 12 comorbidities available in the ASCS [10, 13]. The comorbidities that were included were cerebrovascular disease, CHF, chronic obstructive pulmonary disease (COPD) or asthma, hypertension, diabetes mellitus, IHD, peripheral vascular disease (PVD), CKD, connective tissue disease, solid organ malignancy, leukaemia and lymphoma. These conditions were considered present if they occurred at any point after inclusion in the ASCS database on patient-reported history or medical record review. Seven CCI comorbidities were excluded as data for these were not collected in the ASCS (depression, mild liver disease, severe liver disease, peptic ulcer disease, hemiplegia, presence of human immunodeficiency virus (HIV) and dementia). CCI definitions and adaptations to the ASCS are displayed in Supporting Table 1. Age was not included in CCI calculations, but was included as a covariate in multivariable analysis. The CCI score was calculated for each participant at each study visit. High comorbidity was defined as a CCI score ≥ 4.

Subcategories using the CCI were defined for further analysis of prescribing practices. The cardiac comorbidity subgroup was defined as a physician-recorded diagnosis of IHD, CHF or hypertension, and the pulmonary comorbidity subgroup was defined as a physician-recorded diagnosis of COPD or asthma.

Health-related quality-of-life data (HRQoL) were collected at each study visit using the Short-Form 36 survey (SF-36), which is divided into physical and mental component summaries, with lower scores representing increasing limitations of physical and mental health. The Scleroderma Health Assessment Questionnaire (SHAQ) disability index was also calculated as a measure of physical function, which higher scores indicating worse physical function.

### 2.1. Statistical Analysis

Characteristics were presented as mean (standard deviation) for normally distributed continuous variables and median (interquartile range) for non-normally distributed data. Categorical variables were presented as a number (percentage). The Student's *t*-test was used to compare normally distributed continuous variables and the Wilcoxon rank-sum test was used for comparisons of non-normally distributed continuous variables, while the chi-square test was applied to compare categorical variables across groups.

Survival analysis for time from PAH diagnosis until all-cause mortality was performed using Kaplan–Meier survival curves with log-rank *p* values and both univariable and multivariable Cox proportional hazards models. Variables for inclusion in multivariable survival models were chosen a priori based on clinical justification and previous literature. Statistically significant variables were defined as *p* ≤ 0.05. Statistical analyses were performed using STATA® Version 17.0 (StataCorp, College Station, TX, USA) for Windows.

## 3. Results

### 3.1. Comparison of Characteristics Between SSc-PAH Patients With High and Low Comorbidity Status

There were 2004 patients who met American College of Rheumatology/EULAR criteria for SSc enrolled in the ASCS at the time of our analysis. Of these patients, 238 (11.8%) had RHC-confirmed SSc-PAH. In comparison with SSc patients without PAH, patients with SSc-PAH were overall older at SSc diagnosis (mean (SD) age of 50.3 (14.4) vs. 46.3 (14.3) years, *p* < 0.001). However, they were similar in sex and proportion of diffuse vs limited disease. Patients with SSc-PAH had a significantly higher prevalence of ANA centromere staining (53.2% vs. 45.2%, *p*=0.02). Meanwhile, patients with SSc without PAH had a significantly higher frequency of Scl-70 antibody (10.5% vs. 15.6%, *p*=0.04). The proportion of patients with RNA polymerase 3 and anti-ribonucleoprotein particle A (anti-RNP) was similar between both groups. Patients with SSc-PAH had a higher incidence of calcinosis (55.1% vs. 42.8%, *p* < 0.001) and digital ulcers (54.9% vs. 44.6%, *p*=0.003).

In terms of internal organ manifestations, there was a higher frequency of ILD in patients with PAH compared to those without (106 (45.7%) vs. 454 (26.2%), *p* < 0.001). Patients with SSc-PAH had a higher frequency of dysphagia (167 (72.6%) vs. 1084 (62.5%), *p*=0.003), although there was no significant difference between the groups for gastroesophageal reflux disease (GORD) and gastric antral vascular ectasia (GAVE). There was no significant difference in other clinical manifestations of SSc.

With regard to HRQoL, patients with SSc-PAH had a significantly lower physical health component score on SF-36 (26.6 (8.4) vs. 33.7 (12.2), *p* < 0.001), as well as a higher SHAQ mean score (4.3 (2.8–6.0) vs. 3.2 (1.5–4.8), *p* < 0.001). The mental component of the SF-36 was not significantly different between the two groups. Characteristics of the whole cohort are presented in Table 1.

### 3.2. Comorbidities in SSc-PAH

A comparison of comorbidities in patients with SSc-PAH vs SSc without PAH using the CCI is shown in Table 2. Patients with SSc-PAH had significantly higher CCI score (3.0 (2.0–4.0) vs. 2.0 (1.0–3.0), *p* < 0.001) and were more likely to be highly comorbid (CCI ≥ 4); 30.3% vs. 18.6%, *p* < 0.001. There was no significant difference for the individual CCI comorbidities of cerebrovascular disease, diabetes mellitus, COPD or asthma, CKD, connective tissue disease or malignancy (solid organ, leukaemia, lymphoma). Patients with SSc-PAH had significantly more cardiovascular disease, including hypertension (61.6% vs. 46%, *p* < 0.001), IHD (27.6% vs. 12.2%, *p* < 0.001), CHF (8.6% vs. 5.2%, *p*=0.04) and PVD (15.4% vs. 7.7%, *p*=0.001).

Within the cohort of SSc-PAH patients, a comparison of patients who were highly comorbid (CCI ≥ 4) and those who were not (CCI ≤ 3) is shown in Table 3. There were no significant differences in clinical characteristics between the groups, including age, sex, proportion of limited vs diffuse disease and autoantibody profile. Similarly, there was no significant difference in cutaneous manifestations or internal organ manifestations between the two groups.

### 3.3. PAH Treatment, Including Medication Class and Prescribing Practices

In the whole cohort of patients with SSc-PAH, 47.9% of patients received combination therapy, 47.0% of patients received monotherapy, and 5.1% of patients received no therapy; *p*=0.10 (Table 3). Among PAH treatments, ERAs were most commonly prescribed at 84.2%, then PDE-5 inhibitors (57.7%) and finally prostanoids (9.4%). There was no significant difference in treatments received by those with high or low comorbidity, with similar proportions receiving combination (24/38, 63.2% vs. 88/196, 44.9%, *p*=0.10) or monotherapy (12/38, 31.6% vs. 98/196, 50%, *p*=0.10).  Table 4 displays subgroup analyses of treatments in SSc-PAH patients with cardiac comorbidity, pulmonary comorbidity or SSc-related ILD, compared to those without. There was no statistically significant difference in prescription of no therapy, monotherapy or combination therapy in patients with concomitant cardiac disease (*p*=0.21), pulmonary disease (*p*=0.32) or those with SSc-related ILD (*p*=0.90). There was no significant difference in HRQoL scores between high and low comorbid SSc-PAH patients.

### 3.4. Survival in SSc-PAH Patients

**Figure 1:**
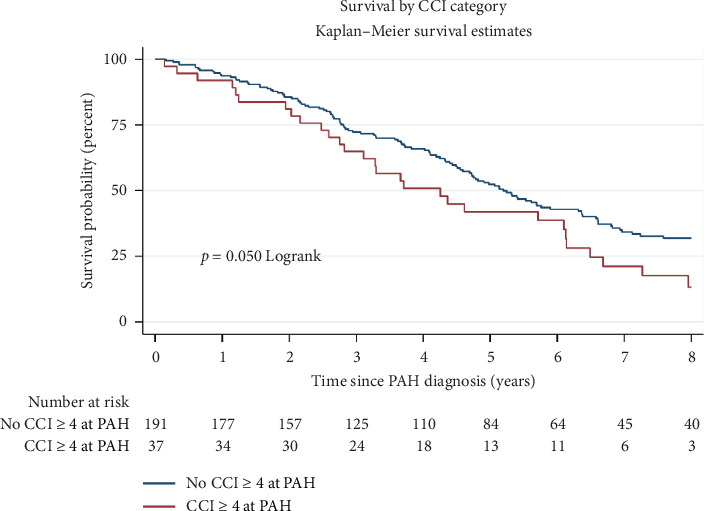
Kaplan–Meier curve of survival from PAH diagnosis to all-cause mortality by multimorbidity status at PAH diagnosis.

When comparing SSc-PAH patients with high comorbidity to those with low comorbidity using K-M survival analysis, there was higher all-cause mortality (*p*=0.05; Figure 1). Univariable survival analysis showed no significant survival difference between high and low comorbidity SSc-PAH patients (Supporting Table 2). In multivariate survival analysis, high comorbidity was associated with poorer prognosis, but this did not reach statistical significance (HR 1.44, 95% CI 0.95 to 2.16, *p* < 0.08). Female sex was significantly associated with improved survival (HR 0.66 (95% CI 0.44–0.98), *p*=0.04), whereas higher mPAP on RHC at PAH diagnosis was associated with worse survival, HR 1.03 (95% CI 1.02–1.05), *p* < 0.001 (Table 5). Patients on combination therapy had better survival compared to those on monotherapy, HR 0.60 (95% CI 0.43–0.84), *p*=0.003.

## 4. Discussion

Using data from the ASCS, our findings reveal several key points regarding treatment practices, HRQoL and survival outcomes in the context of high comorbidity in SSc-PAH. Our results showed improved survival for patients treated with combination therapy, as compared to monotherapy, irrespective of comorbidity status. Importantly, our analyses showed no significant difference in treatments received by highly comorbid patients compared to noncomorbid patients. Both high and low comorbidity patients received monotherapy and combination therapy with similar frequency, highlighting that comorbidity may have played a smaller role in decision-making than initially hypothesised. When broken down into further subgroups of those with coexisting cardiac disease, pulmonary disease or SSC-related ILD, there was again no difference in treatments. This is noteworthy, given concerns about potential ventilation–perfusion mismatch when prescribing combination therapy to comorbid patients, especially SSc-related cardiovascular or pulmonary conditions. Overall, this prescribing pattern may reflect growing confidence in the safety and efficacy of combination therapy [14].

With regard to HRQoL, patients with SSc-PAH exhibited significantly worse physical component scores compared to those without PAH. However, there was no significant difference in HRQoL between SSc-PAH patients with high and low comorbidity scores. This suggests that the presence of PAH itself, rather than the cumulative burden of comorbidities, is the primary driver of reduced physical function in this population.

Multivariate analysis, adjusting for covariates age at SSc diagnosis, female sex, diffuse disease, mPAP, therapy choice and high comorbidity, showed that while high comorbidity was associated with a worse prognosis, it did not reach statistical significance, possibly due to limited sample size and duration of follow-up (Table 5). Larger cohorts may achieve a significant survival difference between groups.

Our results are in line with other large SSc registry data showing improved survival with combination therapy. The survival benefit observed with monotherapy as compared to no therapy, and greater improvement with combination therapy, highlights the importance of treatment in improving outcomes, irrespective of comorbidity status. A Spanish SSc registry study of 76 patients, conducted by Pestana-Fernandez et al., showed improved survival with sequential and upfront combination therapy in SSc-PAH, although upfront therapy did not reach statistical significance [14]. Additionally, a subgroup analysis of the 118 SSc-PAH patients included in the AMBITION trial (2016) showed a reduction in morbidity and mortality of 56% in those receiving upfront combination of ambrisentan and tadalafil compared with monotherapy with either drug [15].

This study has some limitations. Notably, due to the nature of data collection, combination therapy was recorded as ever administered without distinguishing between upfront and sequential combination therapy. Additionally, the ASCS does not collect data on clinicians' rationale behind the selection of PAH therapy class or the decision to use monotherapy vs combination therapy. The prevalence of monotherapy and no therapy within our cohort (47%) may reflect the limited availability of reimbursed combination therapy through the Pharmaceutical Benefits Scheme (PBS) in Australia prior to November 2019. Since the inclusion of dual therapy on the PBS, clinicians have been able to access publicly reimbursed combination therapy for PAH patients who have World Health Organisation (WHO) Class III or IV limitation in physical function. A small number of patients (12, 5.7%) within the ASCS received no PAH therapy. Possible reasons for this include patient preferences, lack of reimbursed access to advanced PAH therapies in Australia prior to 2009 and some patients presenting with PAH that was too advanced and could only be palliated, prior to routine annual screening for PAH which commenced in 2009.

In our cohort, the CCI served as a useful tool to quantify the burden of comorbidities, although it has limitations when applied to SSc populations. While the CCI is a well-established tool for the measurement of comorbidity, it was modified for retrospective application to the ASCS database because not all variables were available for analysis. We were unable to assess the impact of comorbidities, such as dementia, depression, liver disease and peptic ulcer disease, as these variables are not collected in the ASCS. This likely led to an underestimation of the true frequency of highly comorbid patients in our cohort. An additional limitation is that the ASCS is a longitudinal cohort study and registry data can lead to a ‘survivor bias', with more unwell patients being less likely to survive until enrolment in the registry.

## 5. Conclusion

Our study highlights the significant burden of comorbidities in patients with SSc-PAH and their impact on clinical outcomes. In this large cohort of Australian SSc-PAH patients, those with high comorbidity burden tended to have worse survival. Importantly, there was no difference in treatments (monotherapy vs. combination therapy) according to comorbidity status. The findings are in line with current recommendations from the WSPH to deploy upfront treatment with combination therapy in PAH, regardless of their comorbidity status.

## Figures and Tables

**Table 1:** Demographic and clinical characteristics of patients with SSc-PAH vs SSc patients without PAH.

Variable	All patients (*n* = 2004)	PAH (*n* = 238)	No PAH (*n* = 1766)	*p*
*Demographics*				
Female	1711 (85.4%)	196 (82.4%)	1515 (85.8%)	0.153
Caucasian ethnicity	1714 (91.0%)	211 (92.5%)	1503 (90.8%)	0.378
Age at SSc diagnosis (years)	46.8 (14.4)	50.3 (14.4)	46.3 (14.3)	< 0.001
Follow-up (years)	4.5 (1.4–8.9)	4.6 (2.0–8.1)	4.5 (1.3–9.1)	0.676
Diffuse disease	511 (26.0%)	56 (23.7%)	455 (26.3%)	0.404

*Autoantibody profile*				
ANA centromere	895 (46.2%)	124 (53.2%)	771 (45.2%)	0.022
ENA Scl-70	286 (15.0%)	24 (10.5%)	262 (15.6%)	0.041
RNA polymerase	193 (14.3%)	18 (11.1%)	175 (14.8%)	0.210
Anti-RNP	126 (6.6%)	15 (6.5%)	111 (6.6%)	0.950

*Cutaneous and peripheral vascular SSc manifestations*				
Raynaud's phenomenon^∗^	1905 (96.1%)	225 (95.7%)	1680 (96.1%)	0.787
Digital ulcers^∗^	917 (45.9%)	130 (54.9%)	787 (44.6%)	0.003
Calcinosis^∗^	878 (44.3%)	129 (55.1%)	749 (42.8%)	< 0.001

*Renal SSc involvement*				
Scleroderma renal crisis^∗^	72 (3.6%)	7 (2.9%)	65 (3.7%)	0.565

*Gastrointestinal SSc involvement*				
Dysphagia^∗^	1251 (63.7%)	167 (72.6%)	1084 (62.5%)	0.003
GORD^∗^	948 (48.0%)	101 (43.7%)	847 (48.6%)	0.164
GAVE^∗^	217 (10.8%)	28 (11.8%)	189 (10.7%)	0.620

*Lung SSc involvement*				
ILD on HRCT chest^∗^	560 (28.5%)	106 (45.7%)	454 (26.2%)	< 0.001
Shortest 6-min walk distance	411.7 (134.8)	281.0 (114.3)	437.1 (123.3)	< 0.001

*Right heart catheterisation data*				
mPAP (mmHg)^	28.0 (20.0–36.5)	33.0 (28.0–43.0)	20.0 (16.0–27.0)	< 0.001
PVR (Wood units)^	2.9 (2.0–5.0)	4.2 (3.0–6.8)	2.0 (1.3–2.6)	< 0.001
Mean PCWP (mmHg)^	11.0 (8.0–15.0)	11.0 (8.0–14.0)	10.0 (7.0–16.0)	0.986

*Quality of life and physical function*				
SF-36—physical component score^#^	32.9 (12.0)	26.6 (8.4)	33.7 (12.2)	< 0.001
SF-36—mental component score^#^	40.4 (12.4)	39.9 (12.1)	40.5 (12.5)	0.562
SHAQ score^%^	3.3 (1.7–5.0)	4.3 (2.8–6.0)	3.2 (1.5–4.8)	< 0.001

Abbreviations: ANA = antinuclear antibody, ENA = extractable nuclear antigen, FEV = forced expiratory volume, FVC = forced vital capacity, GAVE = gastric antral vascular ectasia, GORD = gastroesophageal reflux disease, HRCT = high-resolution computed tomography, ILD = interstitial lung disease, *n* = number, PAH = pulmonary arterial hypertension, PCWP = pulmonary capillary wedge pressure, PVR = pulmonary vascular resistance, RNA = ribonucleic acid, RNP = ribonucleoprotein particle A, SF-36 = Short-Form 36 survey, SHAQ = Scleroderma Health Assessment Questionnaire, SSc = systemic sclerosis.

^#^Lowest ever score recorded.

^%^Highest ever score recorded.

^^^Highest in those who underwent more than one right heart catheterisation during follow-up.

^∗^Ever recorded from SSc onset.

**Table 2:** Comparison of comorbidities in patients with SSc-PAH vs SSc patients without PAH using the Charlson Comorbidity Index.

Variable	All patients (*n* = 2004)	PAH (*n* = 238)	No PAH (*n* = 1766)	*p*
*Charlson Comorbidity Index*				
Highest CCI score	2.0 (1.0–3.0)	3.0 (2.0–4.0)	2.0 (1.0–3.0)	< 0.001
High comorbidity (CCI ≥ 4)	401 (20.0%)	72 (30.3%)	329 (18.6%)	< 0.001

*Cardiovascular risk factors and comorbidities*				
HTN^∗^	947 (47.8%)	143 (61.6%)	804 (46.0%)	< 0.001
T2DM^∗^	170 (9.2%)	20 (9.1%)	150 (9.2%)	0.964
IHD^∗^	277 (14.0%)	64 (27.6%)	213 (12.2%)	< 0.001
CHF^∗^	99 (5.6%)	18 (8.6%)	81 (5.2%)	0.044
Cerebrovascular disease^∗^	121 (6.6%)	15 (6.8%)	106 (6.5%)	0.870
Peripheral vascular disease^∗^	124 (8.6%)	26 (15.4%)	98 (7.7%)	0.001

*Respiratory comorbidities*				
Asthma/COPD^∗^	600 (30.3%)	75 (32.5%)	525 (30.0%)	0.446

*Rheumatological disease*				
CTD^∗^	2004 (100.0%)	238 (100.0%)	1766 (100.0%)	

*Renal disease*				
CKD^∗^	30 (1.5%)	6 (2.5%)	24 (1.4%)	0.166

*Malignancy*				
Solid organ malignancy^∗^	287 (14.3%)	43 (18.1%)	244 (13.8%)	0.079
Leukaemia^∗^	6 (0.3%)	0 (0.0%)	6 (0.3%)	0.368
Lymphoma^∗^	27 (1.3%)	3 (1.3%)	24 (1.4%)	0.902

Abbreviations: AMI = acute myocardial infarction, CCI = Charlson Comorbidity Index, CHF = congestive heart failure, CKD = chronic kidney disease, COPD = chronic obstructive pulmonary disease, CTD = connective tissue disease, IHD = ischaemic heart disease, *n* = number, LVEF = left ventricular ejection fraction, T2DM = type 2 diabetes mellitus, TIA = transient ischaemic attack.

^∗^Ever recorded from SSc onset.

**Table 3:** Comparison of characteristics of SSc-PAH patients with high (CCI ≥ 4) and low (CCI ≤ 3) comorbidity.

Variable	All patients (*n* = 234)	CCI ≥ 4 (*n* = 38)	CCI ≤ 3 (*n* = 196)	*p*
*Demographics*				
Female	194 (82.9%)	31 (81.6%)	163 (83.2%)	0.812
Caucasian ethnicity	207 (92.4%)	35 (97.2%)	172 (91.5%)	0.234
Age at SSc diagnosis (years)	50.1 (14.3)	52.2 (13.9)	49.6 (14.4)	0.315
Follow-up (years)	4.6 (2.0–8.1)	4.7 (1.9–7.4)	4.6 (2.0–8.2)	0.991
Diffuse disease	54 (23.3%)	8 (21.1%)	46 (23.7%)	0.723

*Autoantibody profile*				
ANA centromere	123 (53.7%)	22 (57.9%)	101 (52.9%)	0.571
ENA Scl-70	23 (10.2%)	5 (13.5%)	18 (9.6%)	0.470
RNA polymerase	18 (11.4%)	2 (7.1%)	16 (12.3%)	0.435
Anti-RNP	15 (6.6%)	2 (5.3%)	13 (6.9%)	0.709

*Cutaneous and peripheral vascular SSc manifestations*				
Raynaud's phenomenon^∗^	221 (95.7%)	37 (97.4%)	184 (95.3%)	0.574
Digital ulcers^∗^	129 (55.4%)	22 (57.9%)	107 (54.9%)	0.732
Calcinosis^∗^	128 (55.7%)	19 (50.0%)	109 (56.8%)	0.443

*Renal SSc involvement*				
Scleroderma renal crisis^∗^	7 (3.0%)	0 (0.0%)	7 (3.6%)	0.237

*Gastrointestinal SSc involvement*				
Dysphagia^∗^	164 (72.6%)	29 (78.4%)	135 (71.4%)	0.386
GORD^∗^	99 (43.6%)	20 (52.6%)	79 (41.8%)	0.219
GAVE^∗^	28 (12.0%)	5 (13.2%)	23 (11.7%)	0.805

*Lung SSc involvement*				
ILD on HRCT chest^∗^	103 (45.2%)	19 (51.4%)	84 (44.0%)	0.410
Shortest 6-min walk distance	279.8 (114.6)	268.3 (98.6)	281.9 (117.4)	0.519

*Right heart catheterisation data*				
mPAP (mmHg)^^^	33.0 (28.0–43.0)	35.0 (28.0–46.0)	33.0 (28.0–43.0)	0.269
PVR (Wood units)^^^	4.2 (3.0–6.7)	4.8 (3.3–7.9)	4.2 (3.0–6.6)	0.379
Mean PCWP (mmHg)^^^	11.0 (8.0–14.0)	12.0 (9.0–15.0)	11.0 (8.0–13.0)	0.121

*Quality of life and physical function*				
SF-36—physical component score^#^	26.5 (8.4)	25.1 (8.0)	26.9 (8.5)	0.251
SF-36—mental component score^#^	39.6 (11.9)	36.7 (11.6)	40.4 (11.9)	0.103
SHAQ score^%^	1.5 (0.9–2.0)	1.6 (1.1–2.1)	1.4 (0.9–2.0)	0.106

*Treatment*				
Class				
Endothelin receptor antagonist^∗^	197 (84.2%)	33 (86.8%)	164 (83.7%)	0.624
PDE-5 inhibitor^∗^	135 (57.7%)	23 (60.5%)	112 (57.1%)	0.699
Prostanoid^∗^	22 (9.4%)	3 (7.9%)	19 (9.7%)	0.728
Therapy				
No therapy	12 (5.1%)	2 (5.3%)	10 (5.1%)	0.105
Monotherapy	110 (47.0%)	12 (31.6%)	98 (50.0%)	0.105
Combination therapy	112 (47.9%)	24 (63.2%)	88 (44.9%)	0.105

Abbreviations: ANA = antinuclear antibody, ENA = extractable nuclear antigen, FEV = forced expiratory volume, FVC = forced vital capacity, GAVE = gastric antral vascular ectasia, GORD = gastroesophageal reflux disease, HRCT = high-resolution computed tomography, ILD = interstitial lung disease, *n* = number, PAH = pulmonary arterial hypertension, PCWP = pulmonary capillary wedge pressure, PDE5 = phosphodiesterase-5, PVR = pulmonary vascular resistance, RNA = ribonucleic acid, RNP = ribonucleoprotein particle A, SF-36 = Short-Form 36 survey, SHAQ = Scleroderma Health Assessment Questionnaire, SSc = systemic sclerosis.

^#^Lowest ever score.

^∗^Ever recorded from SSc onset.

^%^Denotes highest ever score recorded.

^^^Denotes highest in those who underwent more than one right heart catheterisation during follow-up.

**Table 4:** Comparison of PAH treatment in SSc-PAH patients according to the presence of comorbid cardiac disease, pulmonary disease or SSc-related interstitial lung disease.

Cardiac comorbidity^1^ vs. no cardiac comorbidity	All patients (*n* = 234)	Cardiac comorbidity (*n* = 108)	No cardiac comorbidity (*n* = 102)	*p*
No therapy	12 (5.7%)	4 (3.7%)	8 (7.8%)	0.216
Monotherapy	93 (44.3%)	53 (49.1%)	40 (39.2%)	
Combination therapy	105 (50%)	51 (47.2%)	54 (52.9%)	

Pulmonary comorbidity^2^ vs. no pulmonary comorbidity	All patients (*n* = 189)	Pulmonary comorbidity (*n* = 34)	No pulmonary comorbidity (*n* = 155)	*p*

No therapy	12 (6.3%)	2 (5.9%)	10 (6.5%)	0.322
Monotherapy	82 (43.4%)	11 (32.3%)	71 (45.8%)	
Combination therapy	95 (50.3%)	21 (61.8%)	74 (47.7%)	

SSc-related ILD vs. no SSc-related ILD	All patients (*n* = 189)	ILD with FVC < 70% (*n* = 46)	No ILD with FVC > 70% (*n* = 143)	*p*

No therapy	10 (5.3%)	3 (6.5%)	7 (4.9%)	0.903
Monotherapy	85 (45.0%)	20 (43.5%)	65 (45.5%)	
Combination therapy	94 (49.7%)	23 (50.0%)	71 (49.7%)	

Abbreviations: FVC = forced vital capacity, ILD = interstitial lung disease, *n* = number, PAH = pulmonary arterial hypertension, SSc = systemic sclerosis.

^1^Cardiac comorbidity subgroup included those with a diagnosis of angina, acute myocardial infarction, congestive heart failure or hypertension.

^2^Pulmonary comorbidity subgroup included those with chronic obstructive pulmonary disease or asthma.

**Table 5:** Multivariate analysis of factors associated with survival in SSc-PAH.

Variable		Hazard ratio (95% CI), *p*
Female		0.66 (0.44–0.98), *p*=0.040

Age at SSc diagnosis (years)		1.01 (1.00–1.02), *p*=0.145

Diffuse disease		1.25 (0.85–1.82), *p*=0.256

Mean pulmonary artery pressure on RHC (mmHg)		1.03 (1.02–1.05), *p* < 0.001

High comorbidity (CCI ≥ 4) at PAH diagnosis		1.44 (0.95–2.16), *p*=0.084

PAH therapy	No therapy	2.10 (1.02–4.36), *p*=0.045
	Monotherapy	Reference group
	Combination therapy	0.60 (0.43–0.84), *p*=0.003

Abbreviations: CI = confidence interval, CCI = Charlson Comorbidity Index, PAH = pulmonary arterial hypertension, RHC = right heart catheterisation, SSc = systemic sclerosis.

## Data Availability

The data that support the findings of this study are available from the corresponding author upon reasonable request.

## Supporting Information

Supporting Table 1 shows definitions of the CCI variables included with modifications for the ASCS.

Supporting Table 2 displays univariate analysis of variables and their association with survival in patients with SSc-PAH.
