# Supplementary material for: Impact of Comorbidities on Treatments and Outcomes of Systemic Sclerosis–Associated Pulmonary Arterial Hypertension
Source: Can Respir J. 2025 Nov 3;2025:5021789. doi: 10.1155/carj/5021789 (PMC12602041; doi:10.1155/carj/5021789)
Supplement: Supporting Information — Additional supporting information can be found online in the Supporting Information section. [file 5021789.f1.docx]

**Supplementary Table 1: Charlson Comorbidity Index Calculation and adaptation to ASCS cohort**

| **Charlson Comorbidity Index Item** | **ASCS adaptation and definition** |
| --- | --- |
| **Included items** | |
| Cerebrovascular disease | Recorded history of stroke or TIA |
| Congestive heart failure | LVEF<50% on echocardiogram |
| COPD/Asthma | Patient-reported COPD or asthma at each visit |
| Hypertension | Recorded history of hypertension |
| Diabetes | Recorded history of diabetes mellitus |
| Myocardial infarction | Patient-reported angina or myocardial infarction at each visit, or abnormal coronary artery angiogram or angioplasty. |
| Peripheral vascular disease | Recorded history of peripheral vascular disease |
| Connective tissue disease | ACR/EULAR definition of SSc. |
| Chronic kidney disease | Creatinine>265umol/L, dialysis or renal transplantation at each visit |
| Solid organ malignancy | Recorded history of (excluding non-melanoma skin cancer as well as lymphoma/leukaemia as these are included elsewhere) |
| Leukaemia | Recorded history of leukaemia |
| Lymphoma | Recorded history of lymphoma |
| **Excluded items** | |
| Dementia | Not recorded; excluded |
| Depression | Not recorded; excluded |
| Liver disease - mild | Not recorded; excluded |
| Liver disease – moderate or severe | Not recorded; excluded |
| Peptic ulcer disease | Excluded; not recorded independently of other gastrointestinal SSc manifestations |
| Hemiplegia | Not recorded; excluded |
| HIV/AIDS | Not recorded; excluded |

Abbreviations: AIDS (acquired immunodeficiency syndrome), COPD (chronic obstructive pulmonary disease), HIV (human immunodeficiency virus), LVEF (left ventricular ejection fraction), SSc (systemic sclerosis), TIA (transient ischaemic attack), umol/L (micromoles per litre).

### Supplementary Table 2: Univariate analysis of variables and their association with survival in patients with SSc-PAH

| Variable | | Hazard Ratio (95% CI), p | |  |
| --- | --- | --- | --- | --- |
| Demographics | | | |  |
| Female | | | 0.59 (0.40 to 0.85), p=0.005 |  |
| Caucasian ethnicity | | | 1.25 (0.67 to 2.30), p=0.482 |  |
| Age at SSc diagnosis (years) | | | 1.01 (1.00 to 1.02), p=0.209 |  |
| Diffuse disease | | | 1.14 (0.79 to 1.65), p=0.485 |  |
| Autoantibody profile | | | |  |
| ANA centromere | 0.72 (0.53 to 0.98), p=0.038 | | |  |
| ENA Scl-70 | 1.43 (0.85 to 2.41), p=0.173 | | |  |
| RNA polymerase | 1.79 (1.04 to 3.05), p=0.034 | | |  |
| Anti-RNP | 0.75 (0.40 to 1.38), p=0.355 | | |  |
| Charlson Comorbidity Index | | | |  |
| Highest CCI score | | 0.93 (0.83 to 1.05), p=0.262 | |  |
| Multimorbidity (CCI≥4) | | 0.93 (0.67 to 1.30), p=0.686 | |  |
| SSc cutaneous manifestations | | | |  |
| Highest modified Rodnan skin score | | 1.01 (0.99 to 1.02), p=0.322 | |  |
| Raynaud's disease* | | 0.87 (0.39 to 1.98), p=0.745 | |  |
| Digital ulcers* | | 0.95 (0.70 to 1.29), p=0.755 | |  |
| Calcinosis* | | 0.59 (0.43 to 0.80), p=0.001 | |  |
| SSc internal organ manifestations | | | |  |
| ILD on HRCT chest | | 1.78 (1.30 to 2.43), p<0.001 | |  |
| Renal crisis* | | 0.90 (0.40 to 2.05), p=0.809 | |  |
| Dysphagia* | | 0.67 (0.48 to 0.94), p=0.021 | |  |
| GORD* | | 0.74 (0.54 to 1.01), p=0.060 | |  |
| GAVE* | | 0.96 (0.60 to 1.51), p=0.845 | |  |
| Right heart catheterisation data | | | |  |
| mPAP^ | | 1.02 (1.01 to 1.03), p=0.001 | |  |
| PVR (Wood units)^ | | 1.00 (1.00 to 1.01), p=0.687 | |  |
| PCWP^ | | 0.94 (0.91 to 0.98), p=0.001 | |  |
| Treatment | | | |  |
| Class | | | |  |
| Endothelin receptor antagonist* | | 0.37 (0.24 to 0.56), p<0.001 | |  |
| PDE-5 inhibitor* | | 0.74 (0.54 to 1.00), p=0.047 | |  |
| Prostanoid* | | 0.74 (0.45 to 1.22), p=0.241 | |  |
| Therapy | | | |  |
| Monotherapy | | 0.61 (0.30 to 1.21), p=0.158 | |  |
| Combination therapy | | 0.45 (0.22 to 0.90), p=0.024 | |  |
| Quality of life and physical function | | | |  |
| SF-36 – physical component score^#^ | | 1.00 (0.98 to 1.02), p=0.953 | |  |
| SF-36 – mental component score^#^ | | 1.00 (0.99 to 1.02), p=0.564 | |  |
| SHAQ score^ | | 1.03 (0.95 to 1.11), p=0.476 | |  |

Abbreviations: antinuclear antibody (ANA), extractable nuclear antigen (ENA), gastric antral vascular ectasia (GAVE), gastroesophageal reflux disease (GORD), High resolution commuted tomography (HRCT), interstitial lung disease (ILD), phosphodiesterase-5 (PDE-5), pulmonary arterial hypertension (PAH), pulmonary capillary wedge pressure (PCWP), pulmonary vascular resistance (PVR), Ribonucleic acid (RNA), ribonucleoprotein particle A (RNP), Scleroderma Health Assessment Questionnaire (SHAQ), systemic sclerosis (SSc), Short-Form 36 survey (SF-36)

*Denotes ever recorded from SSc onset. ^#^Denotes lowest ever score. ^^^Denotes highest ever score.
